# The Role of Botulinum Toxin Type A in the Clinical Management of Refractory Anterior Knee Pain

**DOI:** 10.3390/toxins7093388

**Published:** 2015-08-25

**Authors:** Barbara J. Singer, Benjamin I. Silbert, Peter L. Silbert, Kevin P. Singer

**Affiliations:** 1 Centre for Musculoskeletal Studies, School of Surgery M424, the University of Western, 35 Stirling Highway, Nedlands, WA 6009, Australia; E-Mails: peter.silbert@gmail.com (P.L.S.); kevin.singer@uwa.edu.au (K.P.S.); 2 Faculty of Medicine, Dentistry and Health Science, the University of Western Australia, 35 Stirling Highway, Crawley, WA 6009, Australia; E-Mail: ben.silbert@gmail.com

**Keywords:** anterior knee pain, muscle imbalance, botulinum toxin type A

## Abstract

Anterior knee pain is a highly prevalent condition affecting largely young to middle aged adults. Symptoms can recur in more than two thirds of cases, often resulting in activity limitation and reduced participation in employment and recreational pursuits. Persistent anterior knee pain is difficult to treat and many individuals eventually consider a surgical intervention. Evidence for long term benefit of most conservative treatments or surgical approaches is currently lacking. Injection of Botulinum toxin type A to the distal region of vastus lateralis muscle causes a short term functional “denervation” which moderates the influence of vastus lateralis muscle on the knee extensor mechanism and increases the relative contribution of the vastus medialis muscle. Initial data suggest that, compared with other interventions for anterior knee pain, Botulinum toxin type A injection, in combination with an active exercise programme, can lead to sustained relief of symptoms, reduced health care utilisation and increased activity participation. The procedure is less invasive than surgical intervention, relatively easy to perform, and is time- and cost-effective. Further studies, including larger randomized placebo-controlled trials, are required to confirm the effectiveness of Botulinum toxin type A injection for anterior knee pain and to elaborate the possible mechanisms underpinning pain and symptom relief.

## 1. Anterior Knee Pain

### 1.1. Pathophysiology of Anterior Knee Pain

Anterior knee pain (AKP), also known as patellofemoral joint pain, is a highly prevalent condition associated with considerable pain and related symptoms, resulting in activity limitation [1]. This condition, often considered to be a sub-set of the broader category of patellofemoral pain, is typically associated with a well-defined group of presenting signs and symptoms, including aching or sharp pain behind the patella, minimal or no effusion, and worsening pain on activities that increase joint loading such as kneeling, squatting, prolonged sitting, and ascending or descending stairs [2]. Anterior knee pain is the most frequently diagnosed orthopaedic condition in young to middle aged adults, representing up to one in six knee complaints presenting to primary health care settings [3]. It is more common in women than men [4]. Some authors have suggested that it may predispose affected individuals to the development of articular degeneration in later life [5,6].

Although the causes of AKP are poorly understood, many potential contributors have been identified [7]. These include: anatomical abnormalities of the patella and/or trochlear groove, mal-alignment of the lower extremity (due to femoral ante-version, external torsion of the tibia, and/or hyper-pronation of the foot), muscle imbalance and soft tissue tightness. The classification system proposed by Witrouw *et al.* [8], which distinguishes mal-alignment from neuromuscular dysfunction, is widely utilized [9]. With respect to the latter category of patellofemoral joint dysfunction, profound disruption of knee extensor force production and motor control can occur due to inhibition of the medial part of the quadriceps muscle (vastus medialis (VM)) related to injury, effusion, or persistent pain [10]. This inhibition results in *relative* over-activity of the lateral component of the quadriceps, the vastus lateralis (VL) muscle, which may lead to abnormal patellar tracking. Coordinated recruitment of the entire quadriceps muscle is required for normal knee joint stability and movement. The view that VM muscle weakness may be an important contributor to AKP receives some support from a long term observational study by Natri *et al.* [11] which found that knee extensor torque was the best predictor of recovery, such that those with the least strength difference between limbs reported the lowest ratings for pain and knee related disability at seven year follow-up [11]. In addition to quadriceps muscle “strength”, abnormalities in the relative timing of muscle activation of the medial and lateral components of this muscle have been the focus of considerable study. A review by Chester *et al.* [12] concluded that data related to the timing of VM *versus* VL muscle activation during knee extension are heterogeneous and that timing anomalies are also seen in pain free individuals. However, in a prospective cohort study of 79 pain-free recruits who undertook a six week strenuous military training program, Van Tiggelen *et al.* [13] reported that delayed onset of electromyographic (EMG) activity of the VM relative to VL muscle was a major risk factor for the 32% of individuals who developed AKP during the training period. More recently, Chen *et al.* [14] reported longer electro-mechanical delays (EMD) in the VM muscle of 26 individuals with AKP compared with age- and gender-matched controls during an isometric maximal quadriceps contraction. Moreover, EMD in the VL muscle of individuals with AKP was shorter than in controls, suggesting that the timing of onset of the components of quadriceps muscle activation may contribute to patellar mal-tracking. Whether this is true motor delay, given their common innervation, or a result of pain inhibition of VM muscle remains equivocal. Excessive lateral patellar tracking associated with muscle imbalance has been suggested to be a major contributor to the development of AKP and joint dysfunction for several decades [15]. Abnormal patella tilt and mal-tracking have been shown to correlate with longer EMD’s of VM muscle and alterations in the ratio of VL:VM activation in people with AKP [16,17].

Lower limb alignment is evaluated by the so-called “Q angle” which has been purported to represent the line of potential force exerted by the quadriceps muscle. The “dynamic Q angle” has been quantified during tasks such as single limb squats and step downs [18,19]. An abnormal motor pattern secondary to contralateral pelvic drop, ipsilateral hip adduction and internal rotation, knee abduction and tibial external rotation with hyper-pronation of the weight-bearing foot, has been postulated to contribute to AKP in some individuals. As a consequence of this finding, a number of investigators have suggested that weakness of the abductor and external rotator muscles of the hip may be implicated in the development of AKP [20], particularly in women [21]. However, the nature of this association has been questioned, with a recent systematic review claiming that disordered hip muscle activation may arise secondary to chronic knee pain and dysfunction, rather than having a causative relationship [22]. Finally, excessive foot pronation inducing medial tibial torsion has also been associated with the development of AKP [7].

### 1.2. Management of Anterior Knee Pain

Conservative treatment for AKP includes a range of options (reviewed by [7,9,23]). Patellofemoral orthoses or taping may be used in an attempt to restore more normal (passive) alignment of the joint. However, Swart *et al.* [24] concluded from their meta-analysis of eighteen studies, only three of which had a low risk of bias, that there is moderate evidence for no additive effect on pain of knee braces compared with exercise therapy alone and conflicting evidence on function. Likewise, only low level evidence exists to support the suggestion that taping the patellofemoral joint may produce greater short-term improvement in pain and function than placebo, and the mechanism for this effect is unclear [25]. The efficacy of some conservative strategies has been shown to be related to the aetiology of AKP, with bracing only relieving symptoms in those individuals accurately classified as “mal-trackers” [26]. Although isolated contraction of VM independent of VL muscle is physiologically not possible, specific “VM muscle strengthening” exercises are a component of most physiotherapy programs, to correct the presumed imbalance in activation of these muscles [9]. Electrical muscle stimulation can achieve VM-specific muscle training and one report of a ten week program of twice daily stimulation in established cases of AKP demonstrated VM hypertrophy and sustained improvement in symptoms, both of which were still present at follow up three and a half years post intervention [27]. Weakness of the hip abductors and external rotators may be corrected with specific muscle re-training [28] and, in selected cases, with external bracing [29,30,31], and there is evidence that knee pain may be reduced by foot orthoses in some individuals [32].

While short term benefits from some non-surgical interventions for AKP such as multi-modal physiotherapy [32], exercise [33] and taping [25] have been reported, the long term effectiveness of these treatments, apart from electrical muscle stimulation [27] has never been convincingly demonstrated. Indicators to predict outcomes of various conservative treatment approaches are also lacking, with a recent systematic review highlighting the paucity of the data on which to base the selection of these interventions [34].

### 1.3. Refractory Anterior Knee Pain

More than two thirds of AKP cases do not respond to conservative therapy with symptoms recurring over time [35,36], and many affected individuals may manage their symptoms by progressively withdrawing from sports participation and restricting pain-provoking activities such as cycling, squatting, kneeling or prolonged sitting. In severe and recalcitrant cases of AKP, surgical intervention has been recommended [23]. However, surgical approaches are generally based on the putative “diagnosis” of tracking abnormalities derived from non-weightbearing CT imaging, despite the fact that this investigation may not accurately predict mal-tracking during physiological loading [37]. Surgical interventions for those with demonstrated patella tracking abnormalities or instability range from passive re-alignment of the patellar tendon with or without lateral capsular release, to the more radical tibial tubercle translocation [23]. The success rate of both conservative and surgical approaches is limited and many individuals experience chronic pain and dysfunction [35,36]. In addition, direct and indirect costs (e.g., loss of productivity), associated with surgical intervention are considerable, as are the risks of increased morbidity. One review comparing outcomes of 344 knees treated with surgery *versus* 403 conservatively treated joints reported that the risk of developing patellofemoral osteoarthritis was doubled in the surgically managed cohort [37]. Consequently, efficacious non-surgical interventions for this prevalent and disabling condition are urgently needed.

## 2. Botulinum Toxin Type A (BoNT-A)

### 2.1. Clinical Uses of BoNT-A

Botulinum toxin type A is one of seven serotypes of the *Clostridium botulinum* neurotoxin. Following intramuscular injection, BoNT-A produces a functional “denervation” via inhibition of acetylcholine release at the neuromuscular junction. In animal models, restoration of muscle function generally occurs within three months, due initially to formation of new terminal nerve sprouts and eventually to recovery of the parent terminal [38]. The use of intramuscular BoNT-A injection to address focal muscle over-activity is well established in the management of focal or axial dystonia in adults [39] and children [40] with acquired brain injury. Treatment of musculoskeletal conditions utilizing intramuscular BoNT-A injection is less commonly reported [41]; however, in the majority of such conditions, BoNT-A has been utilised to relieve muscle spasm and associated pain [42].

### 2.2. The Role of BoNT-A in Managing AKP

To date, a small number of studies have investigated a highly novel treatment of chronic AKP using BoNT-A injection [43,44,45,46,47]. This intervention has involved inducing transient weakening of the distal part of the VL muscle via intramuscular injection of BoNT-A, usually in addition to a standard physiotherapy exercise program to strengthen and improve control of hip and knee musculature. The approach to AKP using BoNT-A injection to the distal third of the VL muscle has much in common with the original application of this neurotoxin to alleviate muscle imbalance causing strabismus, as pioneered by ophthalmologist Dr. Alan Scott [48]. In AKP, the VL muscle is not “overactive” in the same way that a dystonic muscle (e.g., in cervical dystonia) is abnormally active; however due to a loss of balance in the amplitude, and possibly timing, of activation of the VL *versus* the VM muscle, the extensor mechanism may be disrupted and consequently patella tracking may be altered. This represents only one of many potential contributors to AKP, but it is the sub-set of individuals with demonstrable or suspected quadriceps muscle imbalance who have largely been targeted for intervention using BoNT-A to date [43,45,47].

### 2.3. Clinical Trials of Safety and Efficacy of BoNT in AKP

**Figure 1:**
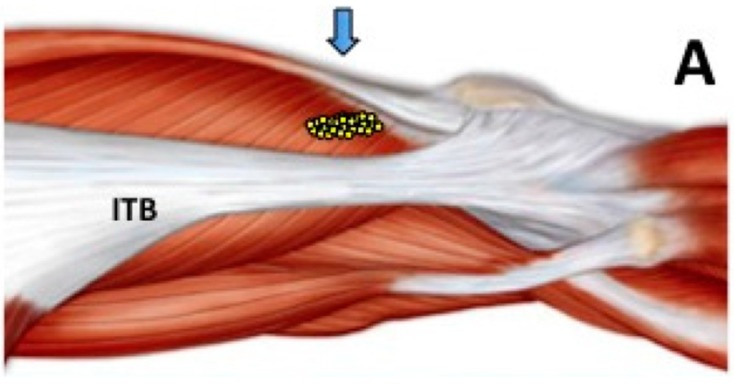

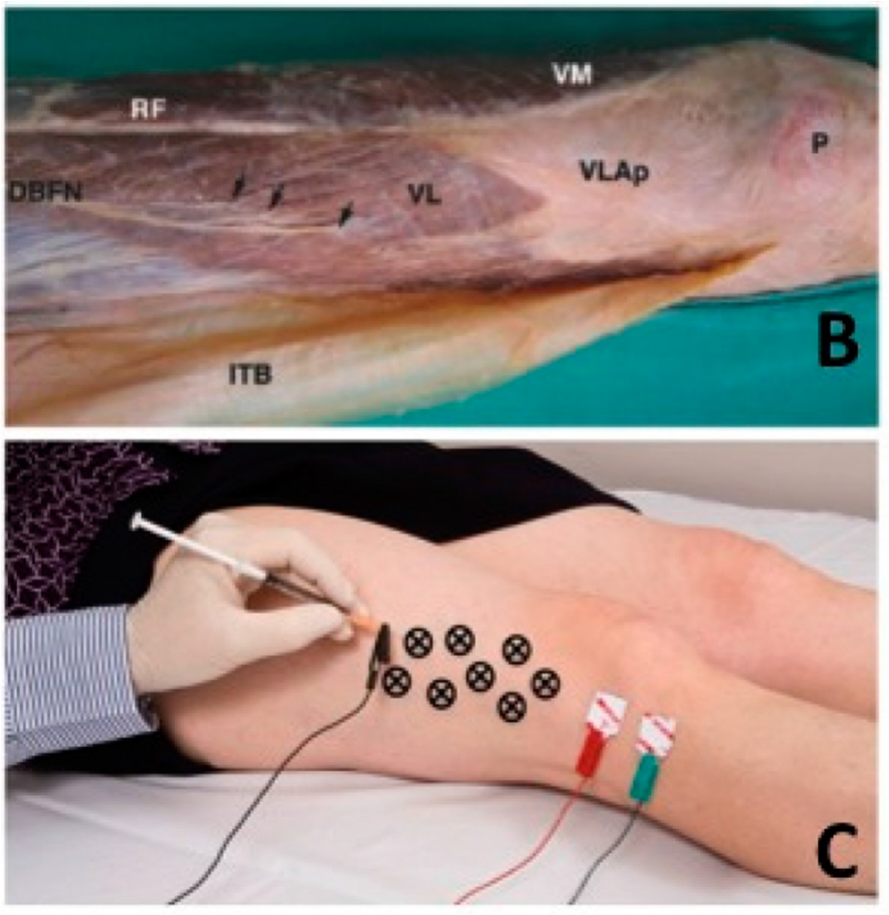
Motor points (arrow) of the distal branch of the femoral nerve (adapted from Botter *et al.* [49]) (**A**). Dissection showing the distal branch of the femoral nerve to Vastus Lateralis (small arrows), with the Iliotibial band (ITB) reflected posteriorly (**B**). As illustrated in (**C**), multiple injection sites, using EMG guidance, were employed to ensure spread of injectate within the distal VL muscle. VLA *p* = vastus lateralis aponeurosis of the knee joint capsule; RF = rectus femoris muscle; VM = vastus medialis; *p* = patella. Reprinted with permission from [45]. Copyright 2011 BMJ Publishing Group Ltd.

**Figure 2:**
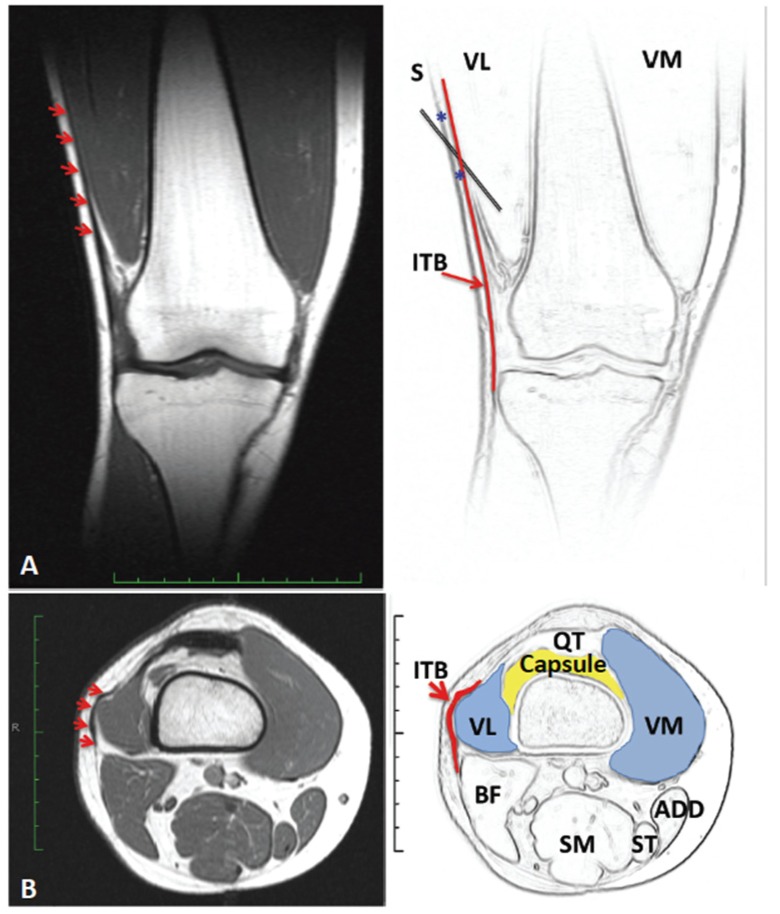
Coronal plane MRI (**A**) to highlight the relationship of the Iliotibial band to the Vastus Lateralis muscle (red arrows). Axial plane MRI to highlight the anatomy at the level of the superior pole of the patella (**B**). Note the proximity of the lateral joint capsule (supra-patella pouch) to the distal end of the VL and the overlying ITB to the VL muscle (red arrows); Legend: VM = vastus medialis, VL = Vastus lateralis, ITB = Iliotibial band, BF = Biceps femoris, SM = Semimebranosus, ST = Semitendinosus, ADD = Adductors, QT = Quadriceps tendon.

In an initial open label pilot study [43] eight female subjects with chronic AKP (mean symptom duration 5 years, range 1–19 years), who had failed conservative management, were injected with BoNT-A (500 U Dysport^®^; Ipsen, Paris, France) to VL muscle and underwent a twelve week individualized home exercise program. As illustrated in Figure 1 and Figure 2, motor points for the distal region of VL muscle are clustered immediately above the tendinous aponeurosis. Needle EMG guidance was used to ensure an intramuscular placement of the BoNT-A injectate. Special care was taken in a few cases with excessive subcutaneous fat to ensure the injectate was placed in the VL muscle. During the angled needle insertion, a second resistance from the overlying fascia of the iliotibial band (ITB) may be perceived (Figure 2). The cohort were relatively young (average age 29 years, range 16–40 years) and had been previously physically active, but were now markedly limited by their AKP. Prior to enrollment in this study, a careful clinical examination of potential subjects was undertaken to exclude those with excessive patellofemoral joint laxity or previous patellar dislocation, BMI > 30, or any contra-indications to BoNT-A injection. CT imaging was used to exclude those with marked joint degeneration. At 12 weeks post injection all subjects demonstrated improvements in extensor isometric force production at 30° flexion (*p* < 0.02) and on a timed stair climbing task (*p* < 0.002). There was also an improvement in knee pain and related symptoms, however the tool utilized (Knee Injury and Osteoarthritis Outcome scale—KOOS) was found to be relatively insensitive to change in this cohort, who subjectively reported improvements in activity limitation and sporting participation that were not captured by the KOOS. Most importantly, few adverse events were reported and these were minor (e.g., soreness around the injection site) which resolved within a few days [43].

**Figure 3:**
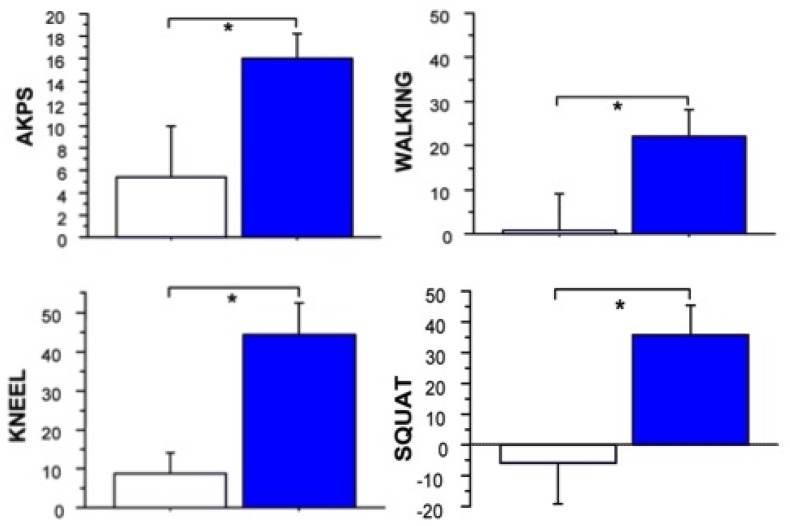
The mean (±SE) change between baseline and 12 weeks post-injection for self-reported knee-related disability and activity induced pain is depicted for BoNT-A injected (blue) *versus* placebo groups (white). Statistically significant differences (*) were seen between groups for pain on kneeling, walking, squatting and Anterior Knee Pain Scale (AKPS) scores. Reprinted with permission from [45]. Copyright 2011 BMJ Publishing Group Ltd.

**Figure 4:**
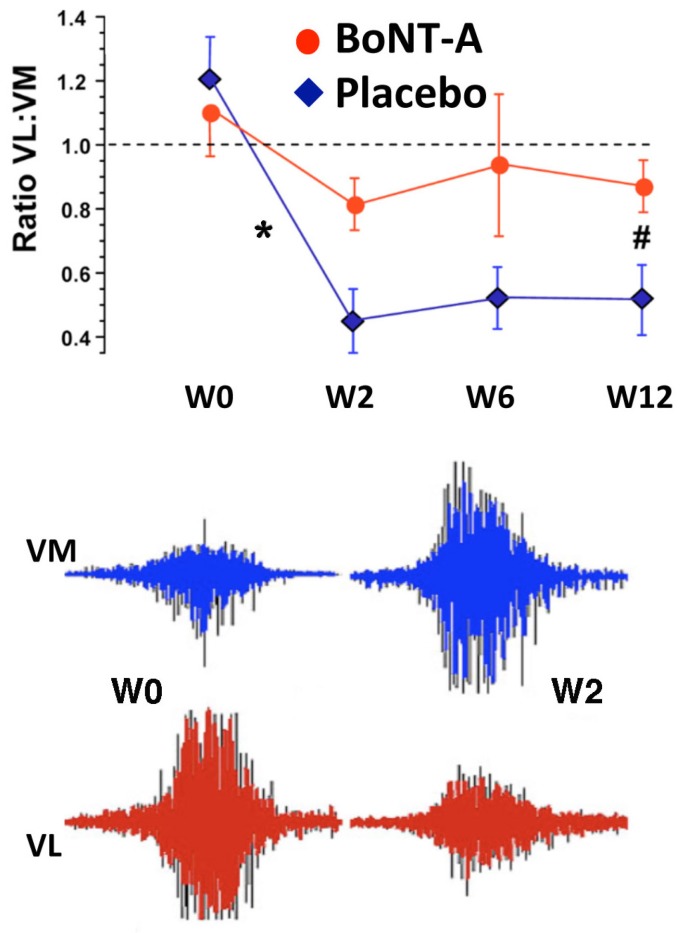
A significant change (*) in VM muscle activation compared to baseline (W0), during static maximal knee extensor contraction at 30° knee flexion, was seen in the BoNT-A injected group only by week 2 (W2) and was sustained at week 12 (W12) (**#**). The dotted line represents a normal ratio of VL:VM activation as assessed from surface EMG. Surface EMG from one subject highlights the relative reversal of VL and VM activation profiles seen in BoNT-A injected subjects only at week 2 (W2) post-injection. Reprinted with permission from [45]. Copyright 2011 BMJ Publishing Group Ltd.

**Figure 5:**
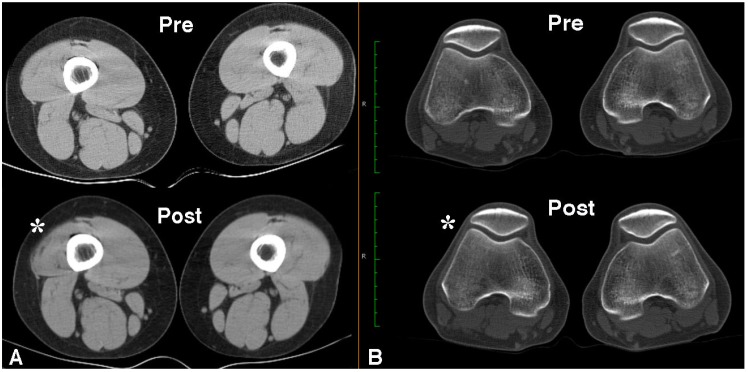
Axial CT images to highlight the anticipated focal atrophy seen in VL muscle at the 12 week follow-up review in the treated limb (*) following BoNT-A injection (**A**). Despite the induced atrophy, no change was recorded in force production nor congruency of the patellofemoral joint during quadriceps contraction at 30° knee flexion (**B**).

In a subsequent double blinded, placebo injection controlled trial by the same group [45], 24 individuals with refractory AKP (mean duration 6 years) were randomly allocated to receive 500 U Dysport^®^ (*n* = 14) or the same volume placebo (*n* = 10) injection to the distal area of the VL muscle, again using EMG guidance to confirm placement. Prior to randomization, in addition to clinical evaluation, VL:VM muscle imbalance was derived from surface EMG recordings during isometric knee extension in 30° flexion. Abnormal VL:VM ratios from surface EMG during a stair climbing task, in addition to self-reported pain (at least 2/10) on provocative tasks such as squatting or stairs, have been shown to be a highly sensitive and specific way of identifying people with AKP [50]. Individuals who did not meet *a priori* criteria for quadriceps imbalance were not included in this trial. All subjects also met the exclusion criteria outlined for the open label pilot study above [43]. Enrolled subjects were again a relatively young group, with a mean age of 29.5 years (range 15–48 years). Mean baseline Anterior Knee Pain Scale (AKPS) scores (BoNT-A group 65/100; placebo 69/100) indicated moderate pain and disability [51]. Following intramuscular BoNT-A or same volume placebo injection and a twelve week individualized home exercise program, BoNT-A injected subjects demonstrated significantly greater improvement in knee pain and disability (measured using the AKPS) compared with those receiving placebo injection (*p* < 0.03), as well as reporting increased participation in sporting and daily living activities. Mean change in the experimental group exceeded the minimal clinically important difference (14 points) [52] for the AKPS (Figure 3). Statistically significant differences from baseline in self-reported pain (assessed using a visual analogue scale) during pain provoking activities were demonstrated only in the group who received BoNT-A injection (Figure 3). The ratio of VL:VM activation during an isometric quadriceps contraction was reversed at 2 weeks post injection (in the BoNT-A group) and remained significantly different from baseline at all time-points post injection (*p* < 0.001) (Figure 4). Subtle changes in ratio for the placebo group may have reflected the influence of the exercise intervention. Static quadriceps muscle force production at 30° knee extension was maintained or improved in BoNT-A injected subjects, despite focal atrophy of the distal component of VL muscle in the treated limb (Figure 5A), supporting the hypothesis that temporarily reducing VL activity would “dis-inhibit” the VM muscle. No measureable change in patellofemoral joint alignment was found from repeated CT assessment at 12 weeks despite the focal distal atrophy of VL muscle (Figure 5B). Subjective and objective improvements in BoNT-A injected subjects were maintained at the 24 week follow-up [45]. In phase two of this study, prior to unmasking of the data, five subjects elected to receive open label injection of BoNT-A, on the basis that they believed their symptoms were unchanged. Upon unmasking of the data, all of these subjects were subsequently found to have received placebo injection in phase one, and most subsequently reported clinically significant improvement following open label BoNT-A injection.

More recently, an open label investigation of twelve men with bilateral symptoms of AKP was undertaken by Chen and colleagues [47]. Subjects with excessive patellar lateral subluxation or tilting evident on Merchant’s view, previous injury or surgery, or severe degeneration of the medial tibia were excluded. The most affected VL muscle was injected with 100U BOTOX^®^ (Allergan plc, Dublin, Ireland) using electrical stimulation to guide injection. The Western Ontario and McMaster Universities Osteoarthritis (WOMAC) Index, as well as eccentric and concentric quadriceps peak torque production at 60°/s using isokinetic dynamometry were measured at four, eight and twelve weeks post injection. Subjects reported improvement in the WOMAC subscales for pain (*p* < 0.014) and function (*p* < 0.029), but not stiffness (*p* < 0.147), for the injected limb. Interestingly, improvements on the WOMAC were also seen in the non-injected limb over the twelve week period, but these were not significant. Improvements in flexion peak torque (eccentric contraction) were seen in both limbs, which may reflect a training effect of repeated testing. The authors reported that quadriceps force production was not impaired by BoNT-A injection of VL, nor were there any adverse events reported post injection [47].

### 2.4. Long Term Effects of BoNT-A Injection in AKP

In addition to these “proof of principle” studies, self-reported outcomes of BoNT-A injection for AKP in the longer term have been investigated [46]. In this study, two groups were surveyed: (a) an unselected cohort of individuals (apart from those under workers compensation or other compensation/insurance schemes) who were referred by orthopaedic surgeons for treatment of refractory AKP using BoNT-A (Dysport^®^ or BOTOX^®^) prior to consideration for surgery. Cases were reviewed from a single private neurology clinic (*n* = 53), none of whom had undergone EMG evaluation to establish quadriceps muscle imbalance prior to injection. In a second series (b), volunteers who had participated in the two previous research studies [43,45] and for whom contact details were available (*n* = 23) were surveyed. Responses were received from 46 of the 53 privately treated patients. Thirty-eight of these reported initial benefit from injection, which was ongoing in 29. Amelioration of symptoms had lasted for a mean ± SD of 25 ± 21 months. Nine individuals reported symptom recurrence after an average of 14 ± 21 months. Ten had undergone knee surgery post-injection, six of whom had not benefitted initially from BoNT-A injection; with only two reporting improvement in their symptoms following surgery. In the second cohort, 19 of 23 previous research participants responded. All had benefited from BoNT-A injection during the initial six month follow up period associated with these studies [43,45]. Symptomatic benefit, with a mean ± SD duration of 44 ± 20 months, had persisted in 15 of the 19 respondents. Four subjects had experienced some return of AKP symptoms (after 21 ± 12 months), although in two cases they were less severe than previously. Two had proceeded to surgical intervention, with one reporting an improvement in symptoms. This audit provides support for the long-term efficacy of intramuscular injection of BoNT-A to remediate chronic AKP which has been unresponsive to conservative management [45]. From these data, of the 29 cases who were considering surgical management for their AKP, the single BoNT-A injection obviated the need for surgery in 25 individuals (86%).

### 2.5. Possible Mechanisms of Effect of BoNT-A Injection in AKP

Intramuscular injection of BoNT-A for chronic AKP is clearly associated with a reduction in self-reported pain and knee related disability, however further research is needed to elucidate the *mechanism(s)* via which BoNT-A injection may induce these improvements. On the basis of the studies reported above, we postulate that improvements in quadriceps motor control result from the opportunity to “unmask” the VM muscle, which is afforded by temporary weakening of VL muscle. The improvement in control of quadriceps activation has a sustained positive effect on recruitment of the knee extensor mechanism and consequently on patellar tracking. However BoNT injection may also contribute an anti-nociceptive effect via other mechanisms, in addition to this mechanistic change.

For instance, some authors have suggested that elevated stress at the cartilage-bone interface may be responsible, in part, for AKP [51]. Wojtys *et al.* [53] have reported that the retinaculum, fat pad, periosteum, and subchondral plate of the patella are all extensively innervated with nociceptors. Peripheral sensitization can occur when there is injury causing joint swelling or tissue disruption. The depolarisation threshold of the articular nerves (which contain A-delta, A-beta and C fibres) may be reduced such that previously non-nociceptive inputs (pressure, motion *etc.*) start to be perceived as painful. In chronic oedema in rodent models, secondary central sensitization has been demonstrated to occur due to progressive hyper-excitability of the spinal nociceptive system [54]. In AKP it is proposed that a combination of mechanical and neural adaptations to abnormal joint stresses may combine to produce chronic joint pain, which is usually activity related, particularly in tasks involving significant patellofemoral loading.

Studies in rat models have shown that injection of BoNT-A can inhibit formalin-induced oedema and glutamate release, subsequently dampening down or preventing spinal sensitization [55]. In addition, in capsaicin induced rat models of interstitial cystitis, BoNT-A induced blockade of substance P (SP) [56] and calcitonin gene related protein (CGRP) [57,58] release has been demonstrated. Neuropeptides such as SP and CGRP are thought to be the main mediators of neurogenic inflammation [58]. Intra-muscular injection of BoNT-A has also been shown to inhibit release of a number of other cytokines and neuropeptides which could reduce central and peripheral sensitization [59,60,61]. *In vivo* models of BoNT-A induced anti-nociception in humans have mostly used sub- or intra-dermal injection. In control subjects, intra-dermal BoNT-A injection has not been shown to have an effect on thresholds for pain induced by electrical stimulation, hot/cold, mechanical pressure or capsaicin [62,63,64,65]. However, intradermal injection of BoNT-A in individuals with neuropathic pain secondary to diabetic neuropathy has been shown to produce significantly greater self-reported pain relief than placebo injection [66]. Hence the response to BoNT-A may depend on the state of the “host nervous system”. Another possibility is that, following injections into the distal third of the VL muscle, including the distal musculo-tendinous portion of the muscle (Figure 1 and Figure 2), some diffusion of BoNT-A may have occurred into adjacent structures, and even into the joint. Spread of injected toxin across muscle fascia (into adjacent muscles) has been demonstrated in animal models [67,68]. If this were the case, pain relief due to blockade of a range of nociceptors (SP, CGRP) and excitatory neurotransmitters (most notably glutamate) may have contributed to the reported symptom relief in this population with chronic AKP. In a series of randomized controlled investigations examining the effect of *intra-articular* injection of BoNT-A for refractory osteoarthritis or pain associated with joint arthroplasty, researchers have reported short term benefit with regard to pain reduction and self-reported improvement in function and quality of life [69]. It would not be unreasonable therefore to speculate that the possible leakage, in some cases, of BoNT-A into the superior aspect of the knee joint capsule (refer Figure 2), together with the focal effect of the drug on muscle mechanics and pain mechanisms, may contribute to the clinical effects seen following BoNT-A injection in cases with AKP.

### 2.6. Clinical Algorithm for the Use of BoNT-A in AKP

**Figure 6:**
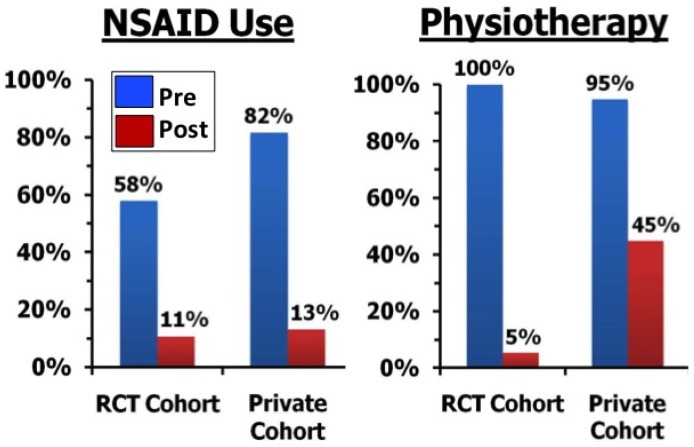
Percentage of cases from an audit survey [46] who reported using non-steroidal anti-inflammatory drugs (NSAID) and/or physiotherapy prior to and following BoNT-A injection for AKP. In both groups, reliance upon NSAID and physiotherapy was markedly diminished following BoNT-A injection for AKP.

Anterior knee pain is a common condition which primarily affects active people from adolescence to middle age, resulting in significant economic and social costs from pain and activity limitation. In this context, we do not regard injection of botulinum toxin as a “first line” of treatment for people presenting with AKP. However, for the majority of individuals who go on to have recurrent symptoms following initial conservative management [35,36], alternative effective non-surgical treatments are urgently needed to avoid chronicity of pain and activity limitation. Surgery is recognised as a last resort for highly selected cases with severe pain and disability associated with patella mal-tracking and instability [23]. Intramuscular injection of BoNT-A into the distal VL muscle produces reversible dose-related weakness, and can confer a “window of opportunity” to effect a lasting change in the balance of activation between VL and VM muscles, restoring more normal control of knee extension, and thereby contributing to long term symptom relief [43,44,45,46,47]. The audit data reported here [46] support the long term efficacy of intramuscular injection of BoNT-A (providing a mean of two years of symptom relief) to remediate chronic AKP which had been unresponsive to conservative management, even in relatively unselected cases who possibly had a range of contributing factors. In this case series, BoNT-A injection was associated with reduced reliance on pain relieving drugs and physiotherapy attendance (Figure 6), and in many cases, avoided the need for surgery [46]. To date, the only other conservative intervention for AKP to demonstrate long term efficacy is electrical muscle stimulation to the VM muscle [27]. Although BoNT-A injection is relatively costly, it can be argued that the point when the administration of other less evidence based interventions equals the cost of a single BoNT-A treatment is quickly reached, particularly as VL:VM imbalance is commonly demonstrated in this condition [9]. Therefore early consideration of BoNT-A injection in the clinical management of this condition could be considered to be a cost effective strategy to moderate the risk of progressive activity limitation and the development of secondary morbidities [5,6].

The injection process, which can be performed in outpatient clinic settings, is a less invasive procedure than surgery, has a low adverse event profile, and is very time and cost efficient relative to many other existing interventions. Consequently, it is proposed that BoNT-A injection be considered as a preliminary intervention in individuals considering surgery for AKP secondary to confirmed patella mal-tracking or instability disorders to allow effective VM strengthening to be undertaken pre-operatively. In some cases, this strategy may eliminate the need for surgery. Unlike surgery, Botulinum toxin has the advantage that the induced “functional denervation” is reversible [38]. However, as demonstrated by Singer *et al.* [45] and Silbert *et al.* [46], the improvement in pain and knee related disability considerably outlasts the expected three month duration of drug effect and many individuals achieve prolonged symptom relief from a single BoNT-A treatment.

### 2.7. Future Research

Further studies, including larger randomized placebo-controlled trials, are required to confirm the effectiveness of BoNT-A injection for AKP and to investigate the hypothesized mechanisms underpinning pain and symptom relief, including biomechanical and biochemical contributions to clinical outcomes. Sub-grouping of AKP is warranted to aid the administration of appropriate cost effective intervention [7,9,23,32]. The focus of most existing BoNT-A studies in AKP has been on individuals with patella mal-tracking associated with VL:VM imbalance [43,44,45,47]; however symptom relief has also been reported in more complex orthopaedic cases with potentially other contributors to their symptoms [46]. Although this has not been demonstrated to date, subtle changes in patellar tracking may occur following BoNT-A injection of the VL muscle as quadriceps control is restored. Physiological loading studies of the patellofemoral joint to further elucidate this hypothesis [26] would be important to refine eligibility criteria and the indications for BoNT-A injection, and to optimize treatment protocols by exploring the duration of effect and determinants of the BoNT-A dose-response relationship. Given the paucity of evidence supporting the long-term effectiveness of most non-surgical and surgical treatment options for AKP, a single intramuscular BoNT-A injection, with appropriate concurrent clinical management, would appear to offer a cost-effective alternative to remediate this common musculoskeletal condition.

## 3. Conclusions

Intramuscular BoNT-A injection into the distal region of VL muscle is safe, and confers a cost and time effective alternative to both ongoing conservative management and/or surgery for individuals with refractory AKP. A key finding of this original use of BoNT-A is the sustained improvement in knee pain and symptoms, and reduced reliance on therapeutic and pharmaceutical management. From clinical audit data of long term outcomes it is notable that many cases did not progress to a surgical intervention after injection of BoNT-A for their chronic AKP symptoms. Clinical improvement reported in the literature extends well past the expected three month duration of drug effect, and is associated with increased activity and sports participation.
